# Similar outcomes after haploidentical transplantation with post-transplant cyclophosphamide versus HLA-matched transplantation: a meta-analysis of case-control studies

**DOI:** 10.18632/oncotarget.18862

**Published:** 2017-06-29

**Authors:** Zhenyang Gu, Li Wang, Lei Yuan, Wenrong Huang, Meng Li, Lixun Guan, Qingyi Wang, Zhe Gao, Shasha Zhao, Lan Luo, Feiyan Wang, Nan Yang, Daihong Liu, Jon C. Aster, Chunji Gao

**Affiliations:** ^1^ Department of Hematology, Chinese People's Liberation Army (PLA) General Hospital, Beijing, China; ^2^ Department of Pathology, Brigham and Women's Hospital and Harvard Medical School, Boston, MA, USA; ^3^ Department of Hematology and Oncology, Laoshan Branch, No. 401 Hospital of Chinese PLA, Qingdao, China; ^4^ Center for Computational and Integrative Biology, Massachusetts General Hospital, Boston, MA, USA

**Keywords:** post-transplant cyclophosphamide, haploidentical, hematopoietic cell transplantation, HLA-matched, similar outcomes

## Abstract

**Background:**

Outcomes of haploidentical hematopoietic cell transplantation (haplo-HCT) with post-transplant cyclophosphamide (PT-Cy) have greatly improved. It remains unknown whether haplo-HCT with PT-Cy was associated with poor outcomes when compared with HLA-matched HCT. To address this issue, we performed a meta-analysis to compare outcomes of haplo-HCT with PT-Cy with those of HLA-matched HCT.

**Methods:**

A systematic search for case-control studies were performed in PubMed, Embase and Cochrane Library databases. Using a random model, the risk ratios (RRs) and 95% confidence intervals (95% CI) were pooled for the final analysis.

**Results:**

Nine case-control studies including 2258 patients (827 patients in the haplo-HCT with PT-Cy group, 748 controls from HLA-matched related donors (MRD), and 683 controls from HLA-matched unrelated donors (MUD)) met the inclusion criteria. No differences were found between haplo-HCT with PT-Cy and HLA-matched HCT with regard to acute graft-versus-host-disease (GVHD), non-relapse mortality, relapse, progression free survival and overall survival. However, haplo-HCT with PT-Cy was found to be associated with a lower incidence of moderate to severe chronic GVHD (Haplo vs MRD: RR=0.54; 95% CI=0.39-0.75; Haplo vs MUD: RR=0.70; 95% CI=0.56-0.88).

**Conclusions:**

The results of this meta-analysis suggest that haplo-HCT with PT-Cy can achieve comparable outcomes with those of HLA-matched HCT. Haploidentical donors can be a feasible and valid alternative when conventional HLA-matched donors are unavailable.

## INTRODUCTION

Despite rapid progress in development of targeted therapy, many hematologic malignancies remain incurable with conventional or targeted therapies. Allogeneic hematopoietic cell transplantation (allo-HCT) remains an effective treatment for most hematologic malignancies. However, the unavailability of HLA-matched related donors (MRD) and HLA-matched unrelated donors (MUD) has greatly limited the widespread application of allo-HCT. Several alternative donors such as haploidentical related donors, mismatched unrelated donors, and umbilical cord blood, are often used for patients without an HLA-matched donor. With the potential to be almost universally available, haploidentical HCT (haplo-HCT) has been extensively investigated. Early haplo-HCT attempts, either the regimens with or without the extensive *in vivo* or ex vivo T-cell depletion, has been limited by higher rates of graft-versus-host disease (GVHD), non-relapse mortality (NRM), and graft rejection [1, 2], or by higher risks of disease relapse, and slow immune reconstitution [3, 4].

With the development of modern transplant procedures, haplo-HCT that utilize T-cell-replete grafts with the help of antithymocyte globulin [5, 6] or high-dose post-transplant cyclophosphamide (PT-Cy) [7–9] has shown promising results. PT-Cy, which is usually administered on days +3 and +4 after stem cell infusion, can selectively eliminate alloreactive T cells stimulated early after transplant without damaging nonalloreactive regulatory T cells, memory T cells, and hematopoietic progenitor cells [10, 11]. Haplo-HCT with PT-Cy, with the advantages of high donor availability, reduced costs and easy application, is increasing popular all round the world. Acceptable results of haplo-HCT with PT-Cy led to comparisons of this technique with those of HLA-matched HCT [12–23]. However, because of the small sample size, short follow-up periods, patient selection bias of these studies and lack of prospective randomized studies, it remains unclear whether haplo-HCT with PT-Cy was associated with inferior outcomes when compared with HLA-matched HCT. Therefore, we perform a meta-analysis of available studies to compare outcomes of haplo-HCT with PT-Cy with those of HLA-matched HCT.

## RESULTS

### Study selection and characteristics

**Figure 1:**
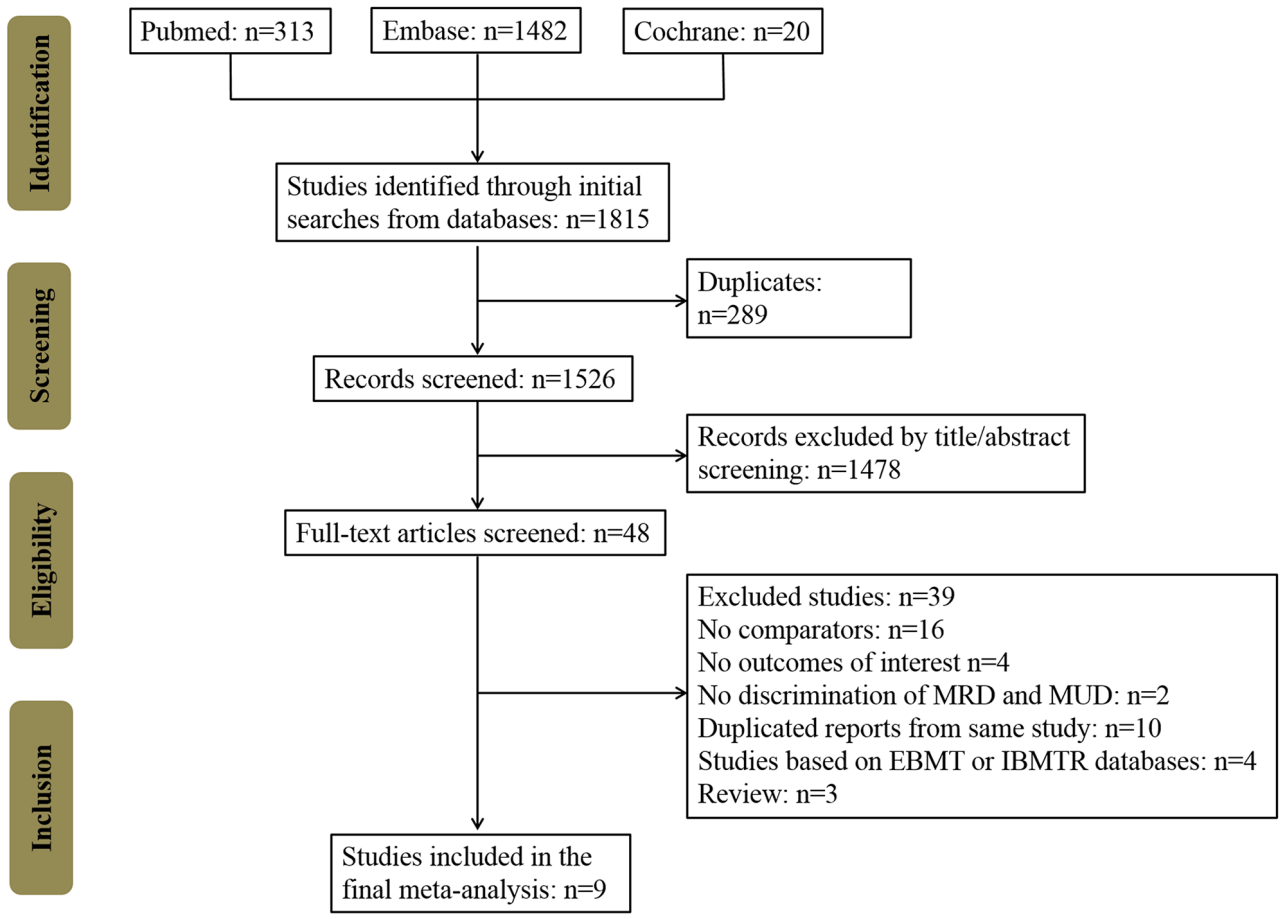
Flow chart of the systematic search used in this study MRD: HLA-matched related donors, MUD: HLA-matched unrelated donors, EBMT: European Group for Blood and Marrow Transplantation, IBMTR: the International Bone Marrow Transplant Registry (IBMTR) databases.

In total, 1815 potentially relevant records were identified in the PubMed, Embase, and Cochrane Library databases (Figure 1). After removing duplicates and screening study titles and abstracts, 1767 non-relevant records were excluded. The full texts of the remaining 48 studies were thoroughly reviewed, resulting in the exclusion of 39 studies that did not meet eligibility criteria. No extra studies were identified during the manual search for the references of these included studies and review articles. Finally, 9 remaining case-control studies included 2258 patients (827 cases in the haplo-HCT with PT-Cy group; 748 controls in the MRD group; and 683 controls in the MUD group) [12–20]. The NOS score of all included studies was >3 (Supplementary Table 1). The characteristics of the included studies are summarized in Tables 1 and 2. Seven studies were performed in the USA, 1 in France and 1 in Italy. Comparisons of haplo-HCT with PT-Cy and MRD and MUD HCTs were all evaluated in six studies. Two studies only compared haplo-HCT with PT-Cy and MUD HCT, and one studies assessed the outcomes between haplo-HCT with PT-Cy and MRD HCT. GVHD prophylaxis in the MRD and MUD groups were mostly conventional regimens, but two studies also used PT-Cy in the control groups.

**Table 1:** Characteristics of included studies, comparing outcomes of Haplo-HCT with PT-Cy with that of HLA-matched transplantation

First author	Patients	Graft source	Conditioning regimen	GVHD prophylaxis		
				Haplo	MRD	MUD
Armin Rashidi[12]	Patients with AML	Haplo: PBSC MUD: PBSC	Haplo: MAC (44%): Flu/TBI; RIC (56%): Flu/Cy/TBI, Flu/Mel; MUD: MAC (44%): Bu/Cy, Cy/TBI; RIC (56%): Flu/Bu;	Tac + MMF + PT-Cy		Tac + MTX ± ATG; Tac + MTX+ MMF± ATG
Didier Blaise[13]	Patients with hematological malignancies	Haplo: PBSC (87%) > BM (13%); MRD: PBSC MUD: PBSC (95%) > BM (5%);	Haplo: RIC: Flu/Cy/TBI, Flu /Bu/Cy, Flu /Bu/Thiotepa; MRD: RIC: Flu /Bu; MRD: RIC: Flu /Bu;	CsA + MMF + PT-Cy	ATG + CsA	ATG + CsA; ATG + CsA + MMF
Antonio Di Stasi[14]	Patients with AML/MDS	Haplo: BM (97%)> PBSC (3%); MRD: PBSC (97%) >BM (3%); MUD: PBSCT (54%) > BM (46%);	Haplo: MAC/RIC: Flu/Mel/thiotepa; MRD: MAC/RIC: Flu/Mel; MUD: MAC/RIC: Flu/Mel;	Tac+ MMF+ PT-Cy	Tac + mini-MTX	Tac + mini-MTX + ATG
Asad Bashey[15]	Patients with hematological malignancies	Haplo: BM (55%) > PBSC (45%); MRD: PBSC (99%) >BM (1%); MUD: PBSC (82%) > BM (18%);	Haplo: MAC (40%): Flu /Bu/Cy, Flu/TBI; NMAC/RIC(56%): Flu/Cy/TBI; MRD: MAC (54%), NMAC/RIC (46%); MUD: MAC (51%), NMAC/RIC (49%);	Tac+MMF +PT-Cy	Tac + MTX ± ATG; Tac + MTX +alemtuzumab	Tac + MTX ± ATG; Tac + MTX +alemtuzumab
Anna Maria Raiola[16]	Patients with hematological malignancies	Haplo: BM; MRD: BM (97%) >PBSC (11%); MUD: BM (60%) >PBSC (40%);	Haplo: MAC (77%), RIC (33%); MRD: MAC (55%), RIC (45%); MUD: MAC (72%), RIC (28%);	CsA+ MMF + PT-Cy	CsA + mini-MTX	CsA + mini-MTX +ATG
Lauri M. Burroughs[17]	Patients with relapsed or refractory Hodgkin lymphoma	Haplo: BM; MRD: PBSC; MUD: PBSC;	Haplo: NMAC: Flu/Cy/TBI; MRD: NMAC: TBI, Flu/TBI; MUD: NMAC: Flu/TBI;	Tac+ MMF+ PT-Cy	CsA + MMF; Tac+ MMF;	CsA + MMF; Tac+ MMF;
Melissa Baker[18]	Patients with hematological malignancies	Haplo: PBSC (56%) > BM (44%); MUD: PBSCT (68%) > BM (32%);	Haplo: RIC: Flu/Cy/TBI; MUD: MAC/RIC: Flu/Bu/TBI/, Flu/Bu, Flu/Mel, Cy/TBI, Flu/TBI;	Tac+ MMF+ PT-Cy		Tac + MTX ± ATG; Tac + MMF ± ATG
Shannon R. McCurdy[19]	Patients with hematological malignancies	Haplo: BM; MRD: BM; MUD: BM;	Haplo: NMAC: Flu/Cy/TBI; MRD: MAC: Bu/Cy, Flu/Bu; MUD: MAC: Bu/Cy, Flu/Bu;	Tac + MMF +PT-Cy	PT-Cy	PT-Cy
Sameh Gaballa[20]	Patients with hematological malignancies	Haplo: NA; MRD: NA;	Haplo: MAC: TBI; MRD: MAC: TBI;	Tac+ MMF +PT-Cy	Tac+ MMF +PT-Cy	

GVHD: graft-versus-host disease; haplo: HLA-haploidentical; n: number; MRD: HLA-matched related donor; MUD: HLA-matched unrelated donor; AML: acute myeloid leukemia; PBSC: peripheral blood stem cells; BM: bone marrow; MAC: myeloablative conditioning; NMA: nonmyeloablative conditioning; RIC: reduced-intensity conditioning; Flu: fludarabine; TBI: total body irradiation; Cy: cyclophosphamide; Bu: busulfan; Mel: melphalan; ATG: antithymocyte globulin; PT-Cy: post-transplant cyclophosphamide; MMF: mycophenolate mofetil; Tac: tacrolimus; MTX: methotrexate; CsA: cyclosporine.

**Table 2:** Characteristics of included studies, comparing outcomes of Haplo-HCT with PT-Cy with that of HLA-matched transplantation

First author	Year of publication	Country	Enrollment period	Sample size	Age (years), median(range)	Clinical outcomes: haplo VS MRD and (or) MUD
Armin Rashidi[12]	2016	USA	2010-2015	Haplo: n=52 MUD: n=88	Haplo: 54 (19-73) MUD: 63 (26-74)	No difference for aGVHD (Grade II-IV), aGVHD(Grade III-IV), moderate-severe cGVHD, NRM, relapse and OS.
Didier Blaise[13]	2015	France	2011-2013	Haplo: n=31 MRD: n=47 MUD: n=63 (13 with 1-antigen mismatch)	Haplo: 62 (56-73) MRD: 62 (55-71) MUD: 64 (57-71)	aGVHD (Grade II-IV), Haplo vs MUD, yes; severe cGVHD, Haplo vs MRD yes, Haplo vs MUD, yes; NRM, Haplo vs MUD, yes; PFS, Haplo vs MUD, yes; No difference for aGVHD (Grade II-IV) Haplo vs MRD, aGVHD (Grade III-IV), NRM haplo vs MRD, relapse, PFS haplo vs MRD, OS
Antonio Di Stasi[14]	2014	USA	2005-2012	Haplo: n=32 MRD: n=87 MUD: n=108	Haplo: 52 (20-67) MRD: 60 (24-76) MUD: 62 (21-76)	No difference for aGVHD (Grade II-IV), aGVHD(Grade III-IV), moderate-severe cGVHD, NRM, relapse, PFS and OS.
Asad Bashey[15]	2015	USA	2005-2014	Haplo: n=116 MRD: n=181 MUD: n=178	Haplo: 51 (20-74) MRD: 52 (18-77) MUD: 53 (19-74)	aGVHD (Grade II-IV), Haplo vs MRD yes; moderate-severe cGVHD, Haplo vs MRD yes, Haplo vs MUD, yes; OS, Haplo vs MRD yes; No difference for aGVHD (Grade II-IV) Haplo vs MUD, aGVHD (Grade III-IV), NRM, relapse, FPS, and OS Haplo vs MUD.
Anna Maria Raiola[16]	2014	Italy	2006-2012	Haplo: n=92 MRD: n=176 MUD: n=43	Haplo: 45 (17-69) MRD: 47 (15-69) MUD: 42 (19-66)	aGVHD (Grade II-IV), Haplo vs MRD yes; cGVHD, Haplo vs MRD yes; No difference for aGVHD (Grade II-IV) haplo vs MUD, aGVHD (Grade III-IV), cGVHD haplo vs MUD, NRM, relapse, PFS and OS.
Lauri M. Burroughs[17]	2008	USA	1998-2007	Haplo: n=28 MRD: n=38 MUD: n=24 (5 with 1-antigen mismatch)	Haplo: 32 (14-62) MRD: 33 (17-64) MUD: 28 (20-45)	PFS, Haplo vs MRD yes; No difference for aGVHD (Grade II-IV), aGVHD (Grade III-IV), moderate-severe cGVHD, NRM, relapse, PFS and OS.
Melissa Baker[18]	2016	USA	2011-2014	Haplo: n=54 MUD: n=59 (14 with 1-antigen mismatch, 8 with 2-antigen mismatch)	Haplo: 50.5 (23-73) MUD: 57 (24-72)	No difference for aGVHD (Grade II-IV), aGVHD (Grade III-IV), moderate-severe cGVHD, NRM, relapse, PFS and OS.
Shannon R. McCurdy[19]	2016	USA	2002-2012	Haplo: n=372 MRD: n=192 MUD: n=120	Haplo: 55 (18-75) MRD: 50 (20-66) MUD: 49 (18-65)	No difference for relapse, DFS and OS.
Sameh Gaballa[20]	2015	USA	2007-2014	Haplo: n= 50 MRD: n= 27	Haplo: 49 (21-65) MRD: 49 (25-63)	No difference for aGVHD (Grade II-IV), aGVHD(Grade III-IV), moderate-severe cGVHD, NRM, relapse, PFS and OS.

haplo: HLA-haploidentical; n: number; MRD: HLA-matched related donor; MUD: HLA-matched unrelated donor; aGVHD: acute graft-versus-host disease; cGVHD: chronic GVHD; NRM: nonrelapse mortality; PFS: progression free survival; DFS: Disease-free survival.

### Incidence of GVHD

**Figure 2:**
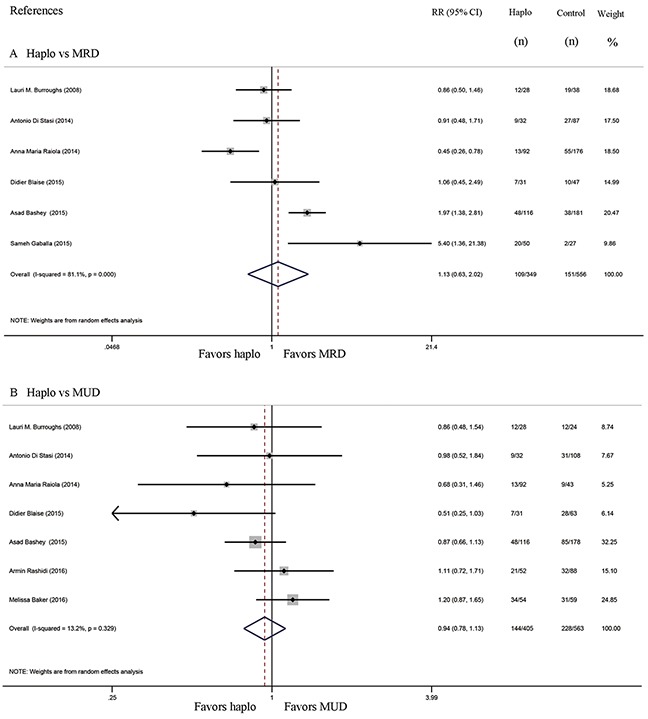
Forest plot and meta-analysis of the approximate 100-day incidence of Grade II to IV aGVHD The incidence rates were similar between halo-HCT with PT-Cy and HLA-matched HCT. Haplo versus MRD (A), Haplo versus MUD (B). aGVHD: acute graft-versus-host disease, HCT: hematopoietic cell transplantation, PT-Cy: post-transplant cyclophosphamide, haplo: HLA-haploidentical, MRD: HLA-matched related donor, MUD: HLA-matched unrelated donor, RR: risk ratio, CI: confidence interval.

**Figure 3:**
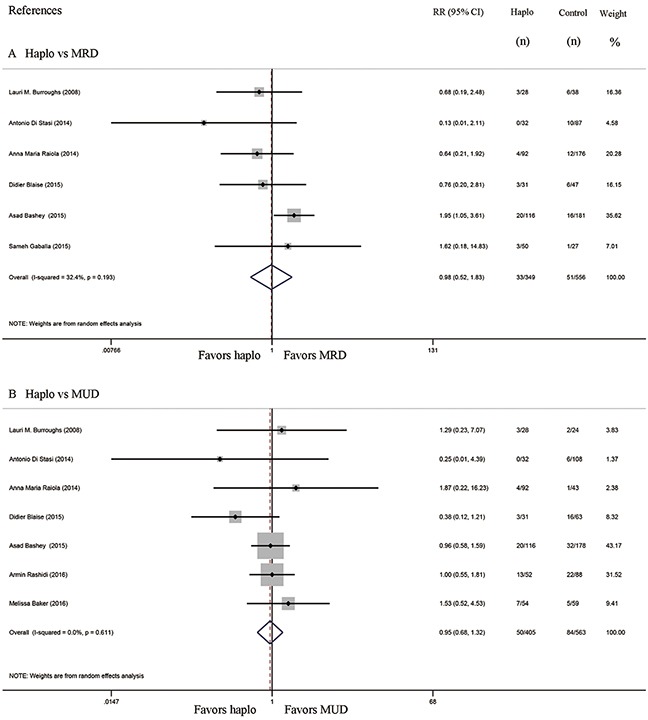
Forest plot and meta-analysis of the approximate 100-day incidence of Grade III to IV aGVHD The incidence rates were similar between haplo-HCT with PT-Cy and HLA-matched HCT. Haplo versus MRD (A), Haplo versus MUD (B). aGVHD: acute graft-versus-host disease, HCT: hematopoietic cell transplantation, PT-Cy: post-transplant cyclophosphamide, haplo: HLA-haploidentical, MRD: HLA-matched related donor, MUD: HLA-matched unrelated donor, RR: risk ratio, CI: confidence interval.

**Figure 4:**
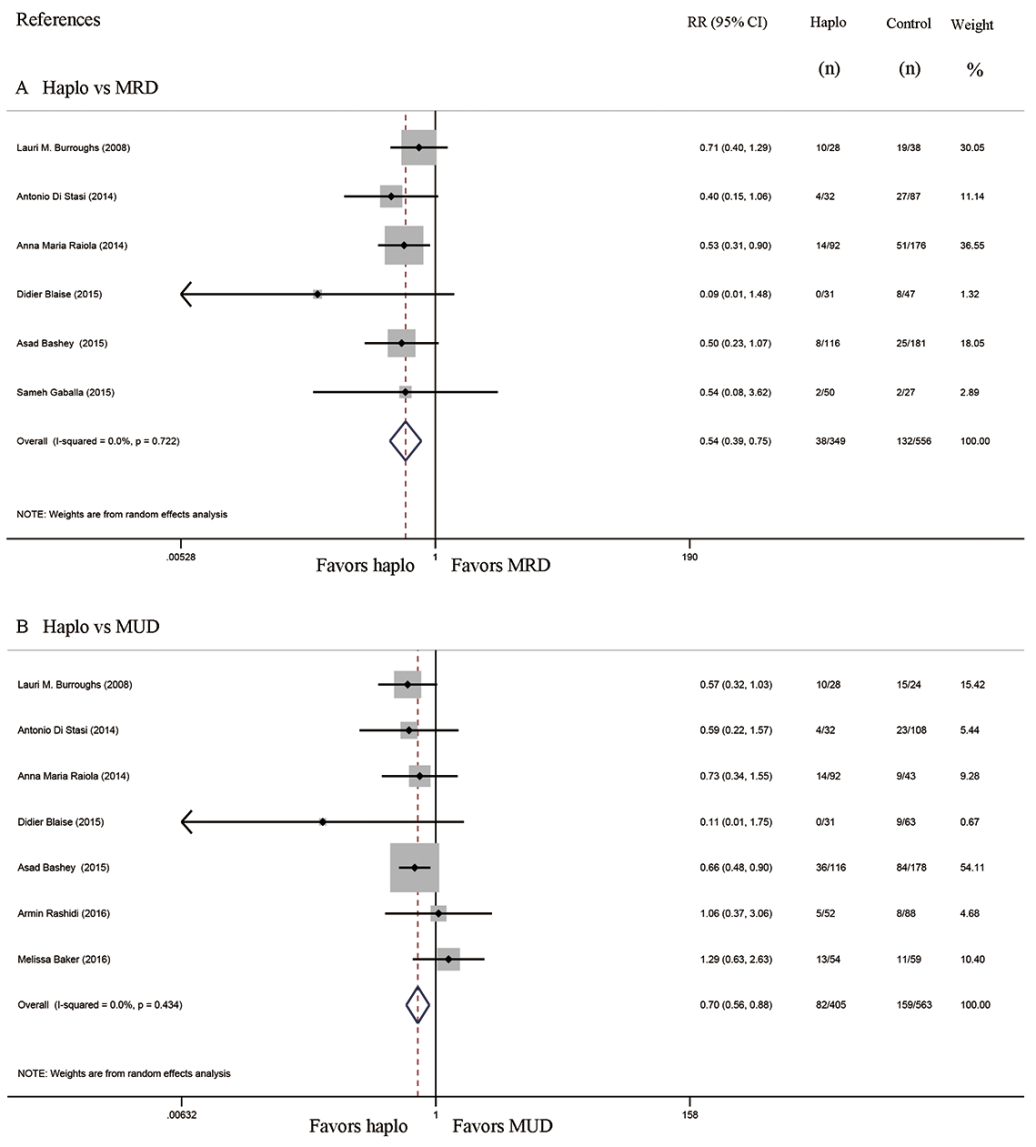
Forest plot and meta-analysis of the approximate 2-year incidence of moderate to severe cGVHD The incidence rate after haplo-HCT with PT-Cy was significantly lower than that of HLA-matched HCT. Haplo versus MRD (A), Haplo versus MUD (B). cGVHD: chronic graft-versus-host disease, HCT: hematopoietic cell transplantation, PT-Cy: post-transplant cyclophosphamide, haplo: HLA-haploidentical, MRD: HLA-matched related donor, MUD: HLA-matched unrelated donor, RR: risk ratio, CI: confidence interval.

Six studies compared the incidence of GVHD between haplo-HCT with PT-Cy and MRD HCT. The approximate 100-day incidence of Grade II to IV aGVHD (RR=1.13, 95% CI=0.63 to 2.02; Figure 2A), and Grade III to IV aGVHD (RR=0.98, 95% CI=0.52 to 1.83; Figure 3A) were similar between halo-HCT with PT-Cy and the MRD group. However, compared with MRD HCT, the approximate 2-year incidence of moderate to severe cGVHD was significantly lower after haplo-HCT (RR=0.54, 95% CI=0.39 to 0.75; Figure 4A). When the data of the seven studies that compared haplo-HCT with PT-Cy and MUD controls were pooled, similar results were obtained. No significant difference was found in the approximate 100-day incidence of Grade II to IV aGVHD (RR=0.94, 95% CI=0.78 to 1.13; Figure 2B), and Grade III to IV aGVHD (RR=0.95, 95% CI=0.68 to 1.32; Figure 3B). Nevertheless, haplo-HCT was associated with a lower 2-year incidence of moderate to severe cGVHD (RR=0.70, 95% CI=0.56 to 0.88; Figure 4B).

Subsequent analysis revealed that three studies included some patients that received one or two HLA-antigen mismatched grafts in MUD control groups [13, 17, 18]. Even when these three studies were excluded, the 100-day incidence of Grade II to IV aGVHD (RR=0.91, 95% CI= 0.74 to 1.12; Supplementary Figure 1), and Grade III to IV aGVHD (RR=0.97, 95% CI=0.67 to 1.42; Supplementary Figure 2) were similar between halo-HCT with PT-Cy and MUD groups. The 2-year incidence of moderate to severe cGVHD in the haplo-HCT with PT-Cy group was still lower than in the MUD groups (RR=0.68, 95% CI= 0.52 to 0.89; Supplementary Figure 3). Further analysis found that bone marrow grafts were more commonly used in the haplo-HCT group. Our study did not include sufficient numbers of patients receiving peripheral blood stem cell (PBSC) grafts to determine the incidence of GVHD in each group. However, in one of our included studies, when only patients that received PBSC grafts were analyzed, the 2-year incidence of moderate-severe cGVHD was still lower than that of HLA-matched controls (p=0.01 for haplo versus MRD, and p=0.002 for haplo versus MUD) [15].

### Hematopoietic recovery

Neutrophil and platelet recovery were compared in seven studies. Because of the different statistical methods used, pooling of data is not possible. Neutrophil recovery were found to be no difference among these 3 groups in three studies [13, 16, 20], whereas four studies showed delayed neutrophil recovery after haplo-HCT with PT-Cy compared to HLA-matched HCT [12, 14, 15, 18]. By contrast, six of these seven studies showed delayed platelet recovery in the haplo-HCT with PT-Cy group as compared to control groups [12–16, 18]. Further analysis revealed the higher percentages of bone marrow grafts being used in the haplo groups of these studies. Bone marrow grafts have been proved to be associated with engraftment delays [24]. It is easy to expect the engraftment delays in haplo-HCT.

### Non-relapse mortality and relapse

**Figure 5:**
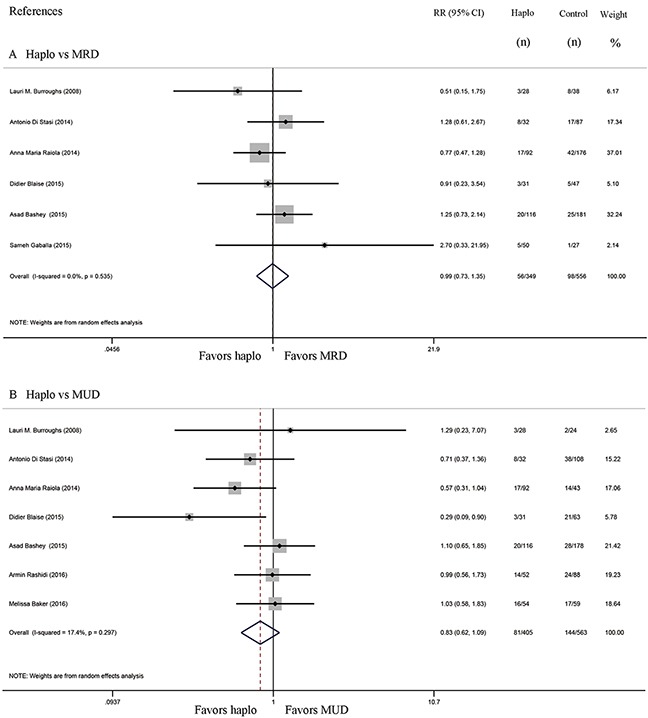
Forest plot and meta-analysis of the approximate 2-year non-relapse mortality It was similar between haplo-HCT with PT-Cy and HLA-matched HCT. Haplo versus MRD **(A)**, Haplo versus MUD **(B)**. HCT: hematopoietic cell transplantation, PT-Cy: post-transplant cyclophosphamide, haplo: HLA-haploidentical, MRD: HLA-matched related donor, MUD: HLA-matched unrelated donor, RR: risk ratio, CI: confidence interval.

**Figure 6:**
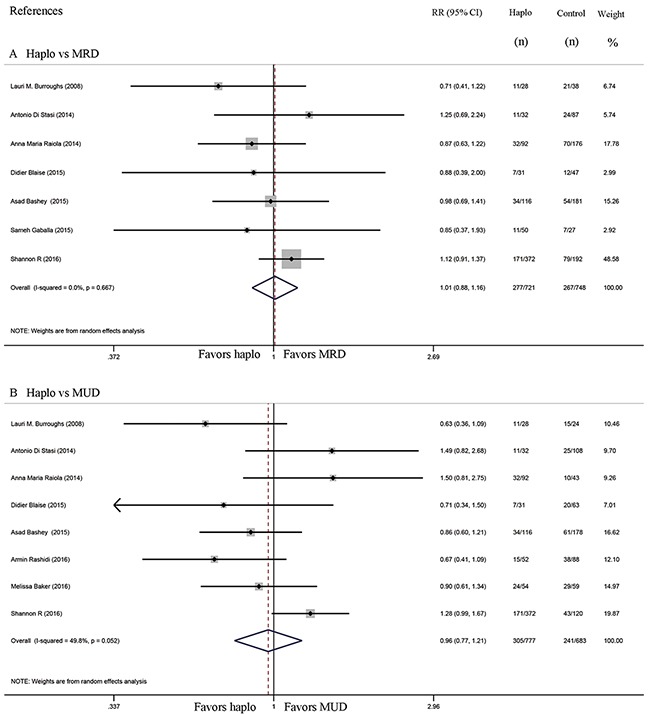
Forest plot and meta-analysis of the approximate 2-year relapse rate It was similar between haplo-HCT with PT-Cy and HLA-matched HCT. Haplo versus MRD **(A)**, Haplo versus MUD **(B)**. HCT: hematopoietic cell transplantation, PT-Cy: post-transplant cyclophosphamide, haplo: HLA-haploidentical, MRD: HLA-matched related donor, MUD: HLA-matched unrelated donor, RR: risk ratio, CI: confidence interval.

Comparisons of NRM between haplo-HCT with PT-Cy and MRD HCT group were reported in six studies, while seven studies compared NRM between haplo-HCT with PT-Cy and MUD HCT groups. The approximate 2-year NRM in the haplo-HCT with PT-Cy group was similar to those in the HLA-matched control group (Haplo vs MRD: RR=0.99, 95% CI=0.73 to 1.35; Figure 5A; haplo vs MUD: RR=0.83, 95% CI=0.62 to 1.09; Figure 5B). Seven studies compared relapse rates between haplo-HCT with PT-Cy and MRD control groups, while comparisons in relapse rates between haplo-HCT with PT-Cy and MUD HCT were reported in eight studies. Again, no difference was found in the comparisons of the approximate 2-year relapse rate (Haplo vs MRD: RR=1.01, 95% CI=0.88 to 1.16; Figure 6A; Haplo vs MUD: RR=0.96, 95% CI=0.77 to 1.21; Figure 6B). Importantly, even when data from only a subset of studies were pooled, where conditioning intensity was similar [12, 16, 20] or more intense [14] in the haplo-HCT with PT-Cy groups, the approximate 2-year NRM and relapse were still similar (see Supplementary Figures 4 and 5).

### Progression free survival and overall survival

**Figure 7:**
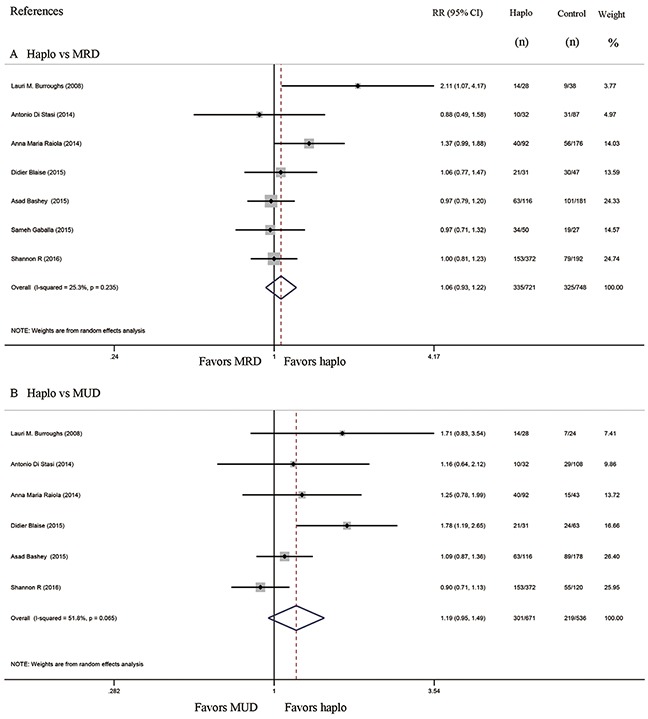
Forest plot and meta-analysis of the approximate 3-year progression free survival. It was similar between haplo-HCT with PT-Cy and HLA-matched HCT Haplo versus MRD **(A)**, Haplo versus MUD **(B)**. HCT: hematopoietic cell transplantation, PT-Cy: post-transplant cyclophosphamide, haplo: HLA-haploidentical, MRD: HLA-matched related donor, MUD: HLA-matched unrelated donor, RR: risk ratio, CI: confidence interval.

**Figure 8:**
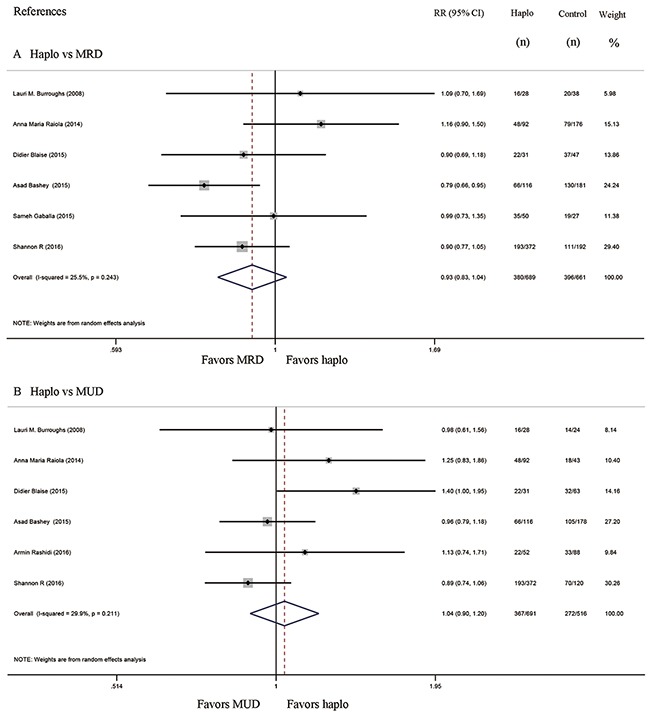
Forest plot and meta-analysis of the approximate 3-year overall survival It was similar between haplo-HCT with PT-Cy and HLA-matched HCT. Haplo versus MRD **(A)**, Haplo versus MUD **(B)**. HCT: hematopoietic cell transplantation, PT-Cy: post-transplant cyclophosphamide, haplo: HLA-haploidentical, MRD: HLA-matched related donor, MUD: HLA-matched unrelated donor, RR: risk ratio, CI: confidence interval.

Seven studies compared progression free survival (PFS) between haplo-HCT with PT-Cy and MRD control group, whereas six compared PFS of haplo-HCT with PT-Cy versus MUD control group. The approximate 3-year PFS in the haplo-HCT with PT-Cy group was similar to those in the HLA-matched control groups (Haplo vs MRD: RR=1.06, 95% CI=0.93 to 1.22; Figure 7A; Haplo vs MUD: RR=1.19, 95% CI=0.95 to 1.49; Figure 7B). Similarly, no difference was found in the comparisons of the approximate 3-year overall survival (OS) between haplo-HCT with PT-Cy group and HLA-matched control groups (Haplo vs MRD: RR=0.93, 95% CI=0.83 to 1.04; Figure 8A; Haplo vs MUD: RR=1.04, 95% CI=0.90 to 1.20; Figure 8B).

## DISCUSSION

Based on all the available case-control studies, our meta-analysis was the first one that compared the clinical outcomes of haplo-HCT with PT-Cy with those after HCTs from MRD and MUD. Our results indicate that with PT-Cy, haplo-HCT can achieve similar outcomes in terms of aGVHD, NRM, relapse rates, PFS and OS, when compared to those seen with HLA-matched HCT in adult patients with hematologic malignancies. Furthermore, the incidence of moderate to severe cGVHD was lower in the haplo-HCT with PT-Cy group. Given the observed similar efficacy and safety, our results suggest that haplo-HCT with PT-Cy is an acceptable alternative to HLA- matched HCT.

In the setting of haplo-HCT, PT-Cy has been shown to be able to preferentially targets the proliferating alloreactive T cells while spares quiescent donor cells, including hematopoietic progenitor cells, regulatory T cells, memory T cells, and non-alloreactive T cells against pathogens and the residue tumor cells. With the ability to selectively deplete of alloreactivity without a prolonged duration of immunosuppression, PT-Cy based GVHD prophylaxis has greatly improved the outcomes of haplo-HCT [7–9]. Here our results showed that with the use of PT-Cy, haplo-HCT can be performed with safety and efficacy which is equivalent to that of HLA-matched HCT. This method that Cy was administered at 50 mg/kg once per day on days 3 and 4 after transplantation was almost exactly identical in all our included studies. Its simplicity reduced medical costs and avoided expensive cell processing, making this procedure can be easily adopted by most transplant centers.

Firstly, because of the heterogeneous diagnosis, there may be some bias in our meta-analysis. But when we further check the composite of underlying diseases in each groups, they were all frequency matched in all our included studies. Secondly, all relevant variables like the percentage of patients in complete remission (CR) before HCT, Disease Risk Index (DRI), European Group for Blood and Marrow Transplantation (EBMT) score, or the Hematopoietic Cell Transplant Comorbidity Index (HCT-CI), were all matched or comparable between these groups of all our included studies. One study also conducted further analysis that only included patients in CR. Clinical outcomes were still similar among these groups [14]. Ideally, we should conduct a subset analysis based the conditioning regimens. It is impossible because of lacking data. Nevertheless, conditioning intensity among these groups was controlled for in most of the included studies. Furthermore, registry-based analyses also revealed that even when the conditioning intensity was similar, haplo-HCT with PT-Cy still showed comparable survival outcomes to those of HLA-matched HCT [21–23, 25].

Only moderate to severe cGVHD were found to be lower in the haplo-HCT with PT-Cy group. This can be partially explained by the higher percentage of BM grafts in the haplo-HCT group [26]. Another possible reason is the high-selective depletion of alloreative T cells by PT-Cy. Although no definite conclusion can be drawn about whether haplo-HCT with PT-Cy is associated with delayed neutrophil and platelet recovery, most studies suggest that hematopoietic recovery in the haplo-HCT group is no better than that of HLA-matched controls [12–16, 18, 20–23].

In the setting of reduced conditioning regimen, lower incidence of NRM is often accompanied by higher risk of relapses. But in the myeloablative setting, relapse rates are more dependent on the risks of underlying diseases. Here, our results showed that despite the lower incidence of moderate to severe cGVHD in the haplo-HCT group, NRM and relapse rates were both similar to those in the HLA-matched groups. First, the reason may be the overall similar conditioning intensity among these three groups. Second, it implies that in the setting of haplo-HCT with PT-Cy, the graft versus tumor effect can be independent of chronic GVHD and can also be as effective as HLA-matched HCT. Lower incidence of moderate to severe cGVHD in the haplo-HCT group can translate into much less need for systemic immunosuppressive therapy, which ultimately helps to reduce relapse rates.

Several inherent limitations existed in this meta-analysis, so our conclusions should be interpreted with caution. First, prospective randomized studies are unavailable, and all data used in our study was all based on retrospective case-control studies. Secondly, only 9 studies were included in this study, and the sample size of some of the included studies was small. Thirdly, although the basal characteristics were not significantly different in most of the included studies, there were still some heterogeneities, including different kinds of hematological malignancies, disease status before HCT, graft sources, and conditioning regimens.

In the absence of randomized trials that compared outcomes between different transplant techniques, our results suggest that clinical outcomes with haplo-HCT with PT-Cy are not inferior to those obtained with HLA-matched HCT using MRD and MUD grafts, and that with PT-Cy, haplo donor can at least work as a feasible and valid alternative to conventional HLA-matched donors. If our results were confirmed in future prospective randomized trials, it shall basically change our current donor selection criterion.

## MATERIALS AND METHODS

### Search strategies

A systematic literature search was performed to identify studies that evaluate the efficacy of haplo-HCT with PT-Cy. The search was conducted up to November 30, 2016 in PubMed, Embase, and the Cochrane Central Register of Controlled Trials databases. The search terms combinations include ‘haploidentical’, ‘haplo-identical’, ‘haplo identical’, ‘haplo transplantation’, ‘haplo transplant’, ‘cyclophosphamide’, ‘Cytophosphane’, ‘Cyclophosphane’, ‘Cytophosphan’, ‘Endoxan’, ‘Neosar’, ‘Procytox’, ‘Sendoxan’ and ‘Cytoxan’. The detailed searches are listed in Supplementary Tables 2, 3 and 4. The language was restricted to English. The references of all identified studies were also manually searched to select relevant articles.

### Selection criteria

All studies that evaluated the outcomes of haplo-HCT with PT-Cy versus those of allo-HCT from HLA-matched related donors or unrelated donors in patients with hematological malignancies were included in our meta-analysis, irrespective of the underlying malignancies of patients, the conditioning regimens, and GVHD prophylactic regimens in HLA-matched allo-HCT control groups. Reports about pediatric patients and studies without discrimination of MUD and MRD were also excluded. The primary outcomes included the incidence of acute GVHD and chronic GVHD, and OS. The secondary outcomes included NRM, incidence of relapse, and PFS. Abstracts with incomplete data were also excluded. In order to avoid reanalyzing patients who had already been reported in other included studies, studies based on the European Group for Blood and Marrow Transplantation (EBMT) or the International Bone Marrow Transplant Registry (IBMTR) databases were also excluded in the final analysis. When multiple reports were published from a single study, only the most recent publication or that with the longest period of follow-up was included.

### Data extraction and quality assessment

Two investigators (Gu and Wang) extracted data independently. All data, including first author of the studies, year of publication, country of origin, period of enrollment, sample size, patient age, conditioning regimens, GVHD prophylaxis, and clinical outcomes, were extracted. Discrepancies were resolved by discussion or by consulting a specialist and final consensuses were reached among all authors. Corresponding authors were also contacted to obtain complete data when necessary.

The quality of all included studies were assessed independently by two authors (Gu and Wang) based on the Newcastle-Ottawa Quality Assessment Scale [27]. This system consists of three factors: patient selection for cases and controls, comparability of the study group and outcome assessment. Studies with poor quality (NOS score<3) were excluded. In case of disagreement, consensus was reached by discussion.

### Statistical analysis

All statistical analyses were performed with statistical software (Stata 12.0, Stata Corporation, College Station, TX). The risk ratio (RR) and relevant 95% confidence intervals (95% CI) was used for pooled dichotomous outcomes. The standardized mean difference (SMD), together with the 95% CI, was used for continuous outcomes. Heterogeneity among studies was evaluated with the I^2^ statistics. Significant heterogeneity among the studies was defined as values of I^2^ > 50% and p value less than or equal to 0.10. To identify sources of heterogeneity, sensitivity analysis was performed to identify the source of heterogeneity. A random effects model was used to conduct the meta-analysis, irrespective of whether heterogeneity existed or not.

## SUPPLEMENTARY MATERIALS FIGURES AND TABLES



## Abbreviations

PT-Cy: post-transplant cyclophosphamide
HCT: hematopoietic cell transplantation
GVHD: graft-versus-host disease
haplo: HLA-haploidentical
MRD: HLA-matched related donor
MUD: HLA-matched unrelated donor
NRM: non-relapse mortality
PFS: progression free survivial
OS: overall survival
AML: acute myeloid leukemia
PBSC: peripheral blood stem cells
BM: bone marrow
MAC: myeloablative conditioning
NMA: nonmyeloablative conditioning
RIC: reduced-intensity conditioning
Flu: fludarabine
TBI: total body irradiation
Cy: cyclophosphamide
Bu: busulfan
Mel: melphalan
ATG: antithymocyte globulin
MMF: mycophenolate mofetil
Tac: tacrolimus
MTX: methotrexate
CsA: cyclosporine.
