# Supplementary material for: Similar outcomes after haploidentical transplantation with post-transplant cyclophosphamide versus HLA-matched transplantation: a meta-analysis of case-control studies
Source: Oncotarget. 2017 Jun 29;8(38):63574–86. doi: 10.18632/oncotarget.18862 (PMC5609944; doi:10.18632/oncotarget.18862)
Supplement: Supplementary file 1 [file oncotarget-08-63574-s001.pdf]

Similar outcomes after haploidentical transplantation with post-transplant cyclophosphamide versus HLA-matched transplantation: a meta-analysis of case-control studies

SUPPLEMENTARY MATERIALS

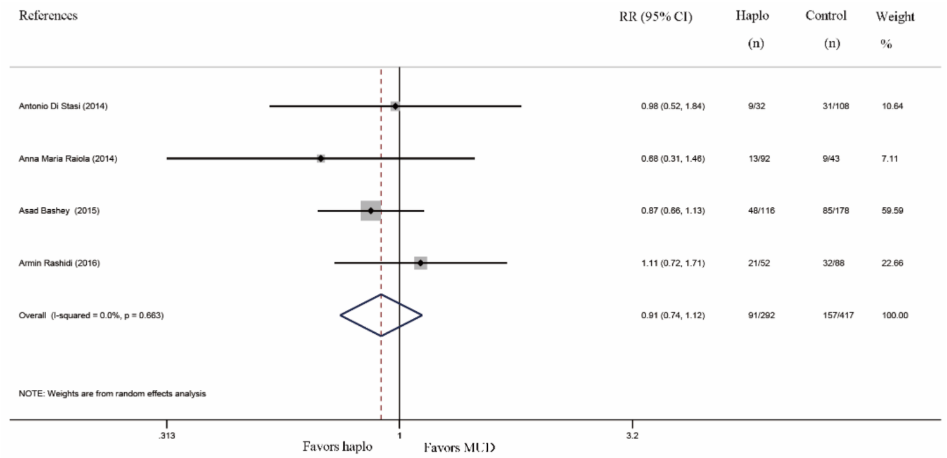

Supplementary Figure 1: Forest plot and meta-analysis of the 100-day incidence of Grade II to IV aGVHD. The incidence rates were similar between halo-HCT with PT-Cy and HCT from MUD when we excluded the three studies that included some patients, who received one or two HLA-antigen mismatched grafts in the MUD control groups. aGVHD: acute graft-versus-host disease, HCT: hematopoietic cell transplantation, PT-Cy: post-transplant cyclophosphamide, haplo: HLA-haploidentical, MRD: HLA-matched related donor, MUD: HLA-matched unrelated donor, RR: risk ratio, CI: confidence interval.

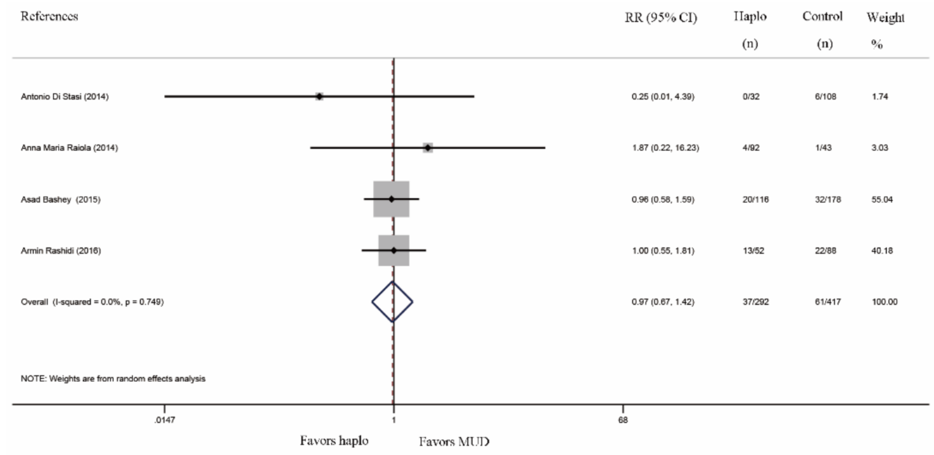

Supplementary Figure 2: Forest plot and meta-analysis of the 100-day incidence of Grade III to IV aGVHD. The incidence rates were similar between halo-HCT with PT-Cy and HCT from MUD when we excluded the three studies that included some patients, who received one or two HLA-antigen mismatched grafts in the MUD control groups. aGVHD: acute graft-versus-host disease, HCT: hematopoietic cell transplantation, PT-Cy: post-transplant cyclophosphamide, haplo: HLA-haploidentical, MRD: HLA-matched related donor, MUD: HLA-matched unrelated donor, RR: risk ratio, CI: confidence interval.

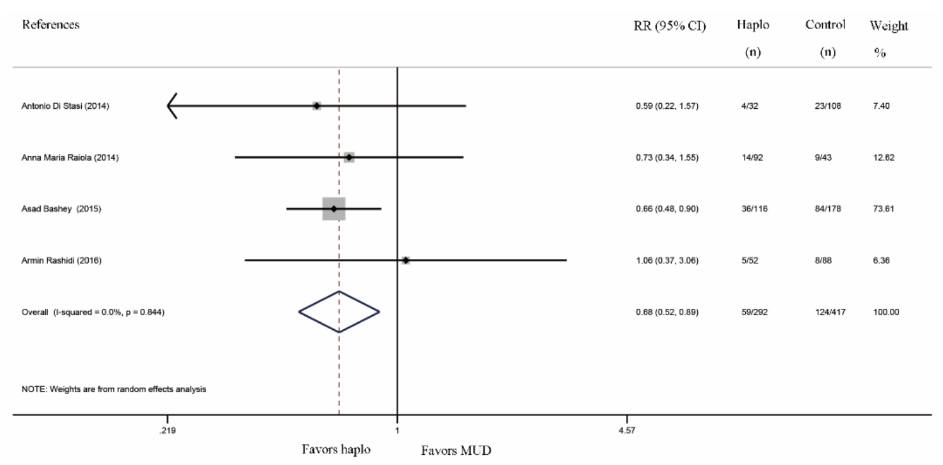

Supplementary Figure 3: Forest plot and meta-analysis of the 2-year incidence of moderate to severe cGVHD. The incidence rates were similar between halo-HCT with PT-Cy and HCT from MUD when we excluded the three studies that included some patients, who received one or two HLA-antigen mismatched grafts in the MUD control groups. cGVHD: chronic graft-versus-host disease, HCT: hematopoietic cell transplantation, PT-Cy: post-transplant cyclophosphamide, haplo: HLA-haploidentical, MRD: HLA-matched related donor, MUD: HLA-matched unrelated donor, RR: risk ratio, CI: confidence interval.

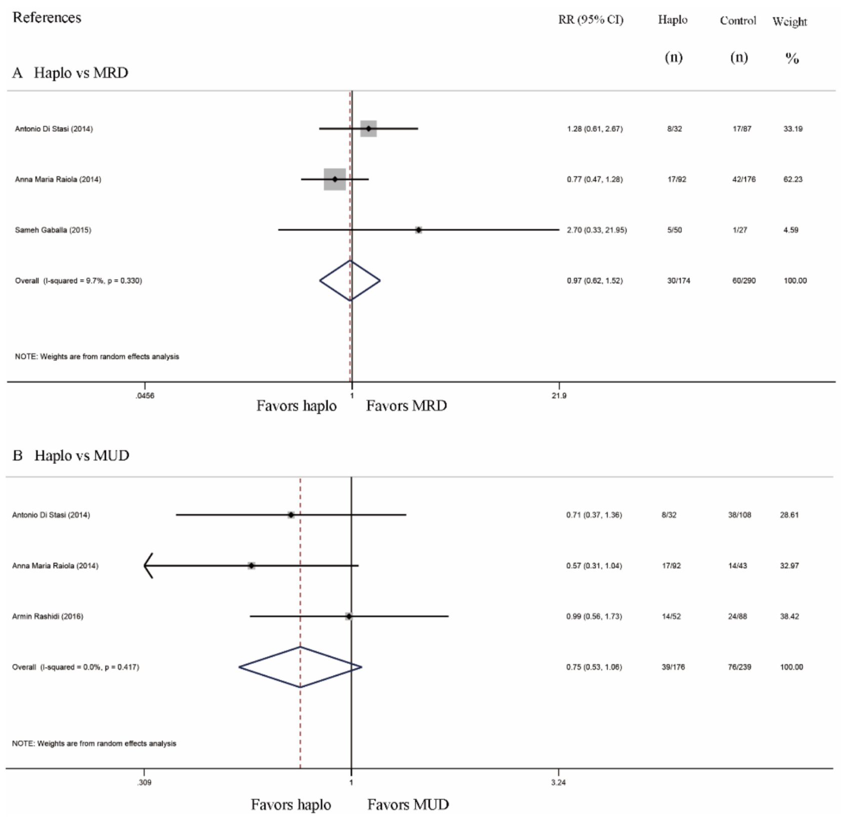

Supplementary Figure 4: Forest plot and meta-analysis of the approximate 2-year non-relapse mortality. It was similar between haplo-HCT with PT-Cy and HLA-matched HCT. Haplo versus MRD **(A)**, Haplo versus MUD **(B)**. HCT: hematopoietic cell transplantation, PT-Cy: post-transplant cyclophosphamide, haplo: HLA-haploidentical, MRD: HLA-matched related donor, MUD: HLA-matched unrelated donor, RR: risk ratio, CI: confidence interval.

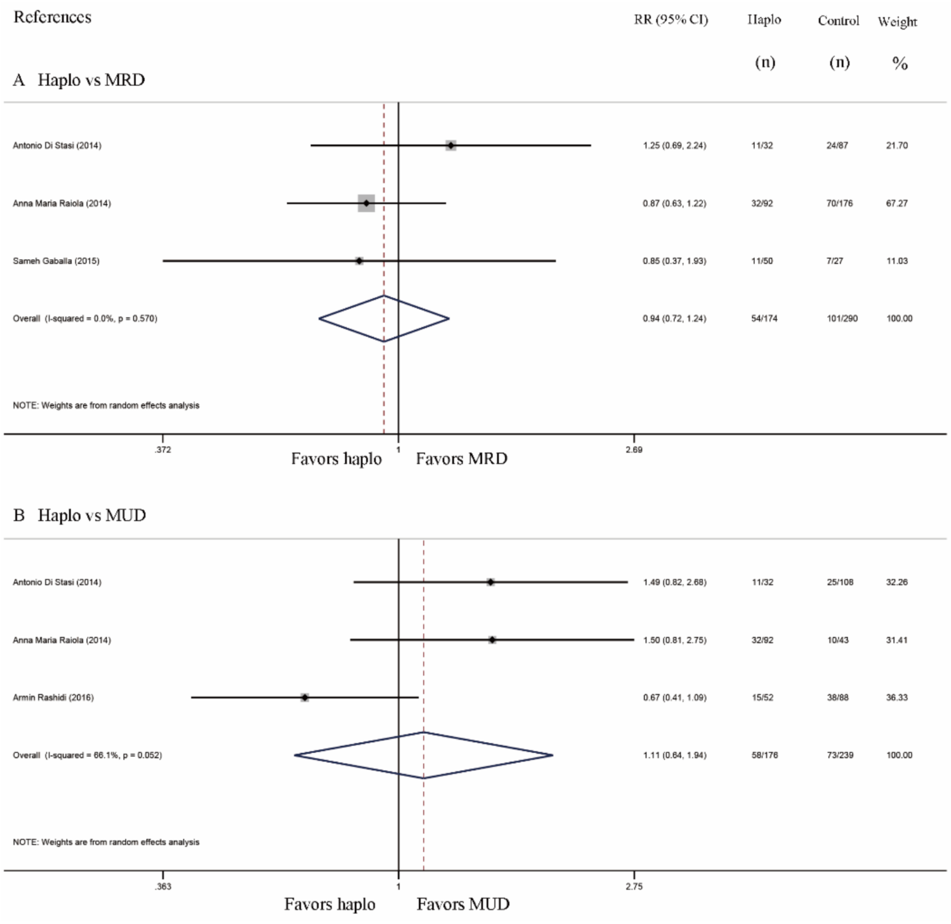

Supplementary Figure 5: Forest plot and meta-analysis of the approximate 2-year relapse rate. It was similar between haplo-HCT with PT-Cy and HLA-matched HCT. Haplo versus MRD **(A)**, Haplo versus MUD **(B)**. HCT: hematopoietic cell transplantation, PT-Cy: post-transplant cyclophosphamide, haplo: HLA-haploidentical, MRD: HLA-matched related donor, MUD: HLA-matched unrelated donor, RR: risk ratio, CI: confidence interval.

Supplementary Table 1: Quality assessment of included non-randomized comparative studies according to the Newcastle-Ottawa quality assessment scale of cohort studies

Author	Representativeness of the cohort	Selection of non-exposed cohort	Exposure ascertainment	Comparable cases and control	Outcome assessment	Duration of follow-up	Adequate follow-up	Adequacy score
**Armin Rashidi[12]**	b	a	a	a	b	b	a	6
**Didier Blaise[13]**	a	a	a	b	b	a	a	7
**Antonio Di Stasi[14]**	b	a	a	b	b	a	a	7
**Asad Bashey[15]**	a	a	a	b	b	a	a	7
**Anna Maria Raiola[16]**	a	a	a	a	b	a	a	7
**Lauri M. Burroughs[17]**	c	a	a	b	b	b	a	5
**Melissa Baker[18]**	a	a	a	b	b	a	a	7
**Shannon R. McCurdy[19]**	a	a	a	b	b	b	a	6
**Sameh Gaballa[20]**	a	a	a	a	b	a	a	7

Representativeness of the cohort: (a*) truly representative, (b*) somewhat representative, (c) selected group, (d) N/R; selection of non-exposed cohort: (a*) same community, (b) different source, (c) N/R; ascertainment of exposure: (a*) secure record, (b*) structured interview, (c) written self-report, (d) N/R; comparable cases and control: (a*) study controls for underlying disease, disease status/risk, (b*) study controls for age, conditioning intensity, donor and so on, (c) study is not controlled; outcome assessment: (a*) independent blind, (b*) record linkage, (c) self-report, (d) N/R; duration of follow-up: (a*) adequate-at least 2 years, (b) not adequate-does not fit (a); adequate follow-up: (a*) complete, (b*)≤20% lost, (c)>20% lost (d) N/R. * represents adequate. Adequacy score was summarized for each study by addition the number of subjects with adequate score (mark with *). Maximal score was seven. Adapted from http://www.endoedu.com/mobile/guideline/NOS_cohort.pdf.

Supplementary Table 2: Search criterion of PubMed (from inception to Nov. 31, 2016)

No.	Query results	Items found
**#18**	Search ((((((haploidentical) OR haplo identical) OR haplo-identical)) OR haplo transplantation) OR haplo transplant) AND ((((((((((cyclophosphamide) OR “Cyclophosphamide”[Mesh]) OR Cytophosphane) OR Cyclophosphane) OR Endoxan) OR Neosar) OR Procytox) OR Sendoxan) OR Cytoxan) OR Cytophosphan)	313
**#17**	Search (((((haploidentical) OR haplo identical) OR haplo-identical)) OR haplo transplantation) OR haplo transplant	2260
**#16**	Search haplo transplantation	364
**#15**	Search haplo transplant	381
**#14**	Search haplo identical	219
**#13**	Search haplo-identical	176
**#12**	Search haploidentical	2026
**#11**	Search (((((((((cyclophosphamide) OR “Cyclophosphamide”[Mesh]) OR Cytophosphane) OR Cyclophosphane) OR Endoxan) OR Neosar) OR Procytox) OR Sendoxan) OR Cytoxan) OR Cytophosphan	66960
**#10**	Search Cytoxan	66865
**#9**	Search Sendoxan	66634
**#8**	Search Procytox	66642
**#7**	Search Neosar	66634
**#6**	Search Endoxan	66689
**#5**	Search Cytophosphan	66634
**#4**	Search Cyclophosphane	66666
**#3**	Search Cytophosphane	66634
**#2**	Search “Cyclophosphamide”[Mesh]	49461
**#1**	Search cyclophosphamide	66634

Supplementary Table 3: Search criterion of Embase (from inception to Nov. 31, 2016)

No.	Query results	Results
#26	'cyclophosphamide'/exp OR cyclophosphamide OR cyclophosphane OR cytophosphan OR endoxan OR neosar OR procytox OR sendoxan OR cytoxan AND ('haplo identical' OR haploidentical OR haplotransplant OR haplotransplantation OR 'haplo transplantation' OR 'haplo transplant')	1482
#25	'haplo identical' OR haploidentical OR haplotransplant OR haplotransplantation OR 'haplo transplantation' OR 'haplo transplant'	4722
#24	'haplo transplant'	27
#23	'haplo transplantation'	15
#21	haplotransplantation	4
#20	haplotransplant	7
#14	haploidentical	4360
#13	'haplo identical'	453
#10	'cyclophosphamide'/exp OR cyclophosphamide OR cyclophosphane OR cytophosphan OR endoxan OR neosar OR procytox OR sendoxan OR cytoxan	196230
#9	cytoxan	4737
#8	sendoxan	111
#7	procytox	64
#6	neosar	197
#5	endoxan	5168
#4	cytophosphan	18
#3	cyclophosphane	216
#1	'cyclophosphamide'/exp OR cyclophosphamide	195967

Supplementary Table 4: Search criterion of Cochrane Library (from inception to Nov. 31, 2016)

No.	Query results	Results
**#1**	MeSH descriptor:[Cyclophosphamide] explode all trees	4108
**#2**	cyclophosphamide:ti,ab,kw (Word variations have been searched)	8279
**#3**	Cyclophosphane:ti,ab,kw (Word variations have been searched)	2
**#4**	Endoxan:ti,ab,kw (Word variations have been searched)	14
**#5**	Neosar:ti,ab,kw (Word variations have been searched)	6
**#6**	Cytoxan:ti,ab,kw (Word variations have been searched)	161
**#7**	#6 or #5 or #4 or #3 or #2 or #1	8659
**#8**	haplo identical:ti,ab,kw (Word variations have been searched)	7
**#9**	haploidentical:ti,ab,kw (Word variations have been searched)	74
**#10**	haplo-identical:ti,ab,kw (Word variations have been searched)	5
**#11**	haplo transplant:ti,ab,kw (Word variations have been searched)	15
**#12**	haplo transplantation:ti,ab,kw (Word variations have been searched)	25
**#13**	#8 or #9 or #10 or #11 or #12	86
**#14**	#7 and #13	20
